# Fuzi and Banxia Combination, Eighteen Antagonisms in Chinese Medicine, Aggravates Adriamycin-Induced Cardiomyopathy Associated with PKA/*β*2AR-Gs Signaling

**DOI:** 10.1155/2018/2875873

**Published:** 2018-09-03

**Authors:** Fengjiao Sun, Yingying Huang, Lili Li, Chao Yang, Pengwei Zhuang, Yanjun Zhang

**Affiliations:** ^1^Tianjin State Key Laboratory of Modern Chinese Medicine, Tianjin University of Traditional Chinese Medicine, Tianjin 300193, China; ^2^Cardiovascular and Cerebrovascular Drugs Research and Development Center, Tianjin Institute of Medical and Pharmaceutical Sciences, Tianjin 300020, China; ^3^Department of School Infirmary, Zhonghuan Information College Tianjin University of Technology, Tianjin 300380, China; ^4^Chinese Materia Medica College, Tianjin University of Traditional Chinese Medicine, Tianjin 300193, China

## Abstract

*Aconite Lateralis Radix Praeparata* (Fuzi) and* Pinelliae Rhizoma* (Banxia) are a combination often used to treat cardiovascular diseases in ancient and modern clinical practice. However, eighteen antagonisms based on traditional Chinese medicine (TCM) theory often abided against such combination therapy. Therefore, exploring whether coadministration of the two herbs can be used in adriamycin- (ADR-) induced cardiomyopathy and clarifying the potential mechanism could help to guide its clinical application. Echocardiography experiments revealed that either Fuzi, Banxia, or their combination had effect on ADR-induced heart dysfunction, while high dose Fuzi exerted positive inotropic effect associated with restored PKA levels. Moreover, low dose Fuzi significantly reduced QT/QTc prolongation, inhibited cardiac apoptosis, and upregulated protein expression of PKA. However, combination of Fuzi and Banxia greatly aggravated QT/QTc prolongation and cardiomyocyte apoptosis in ADR rats compared with each drug alone, which was accompanied by a marked decrease in PKA, pSer346 levels. Similarly, Banxia alone treatment promoted cardiac apoptosis and downregulated protein levels of PKA and pSer346. Additionally, high dose Fuzi treatment also produced proapoptotic effect. Taken together, our study has provided the first direct evidence that combination of Fuzi, a positive inotropic agent, with Banxia promoted cardiac apoptosis in an ADR induced rat model of cardiomyopathy, which may be associated with suppression of PKA/*β*2AR-Gs signaling. This study also provides scientific language for better understanding of the risks and limitations of combination of Fuzi and Banxia in clinical applications.

## 1. Introduction

Duality of toxic and efficacious performance is a nature of drugs, and there could be some undesirable effects in the process of curing disease; traditional Chinese medicine (TCM) is no exception [1]. Clinically, drug compatibility is highly advocated in TCM for thousands of years; it refers to combination of two or more herbs in order to enhance efficacy and simultaneously reduce toxicity [2, 3]. However, if compatibility is improper, it will produce intense toxic reactions or more seriously, it may be lethal; we called this phenomenon “incompatibility” [4].

*Aconite Lateralis Radix Praeparata* (Fuzi) and* Pinelliae Rhizoma* (Banxia) are representative of incompatible pairs recorded in “the eighteen antagonisms”, a special rule for TCM incompatibility in formulas, which suggests strictly compatibility prohibition in clinical prescriptions because mutual antagonism results in an undesirable consequence [3, 5]. However, coadministration of the two herbs has been used in many clinical formulas for several centuries. Indeed, recent statistical analysis reported that 502 classic prescriptions that contain both Fuzi and Banxia, such as Fuzi jingmi Tang, Fuzi San, and Kongxian Wan, are recorded in* Zhongyi Fangji Da Cidian*. In addition, the above studies concluded that combination of Fuzi and Banxia could reduce toxicity and improve pharmacological effect especially for treating cardiovascular diseases [6, 7]. Moreover, this incompatible pair is also widely prescribed in modern clinical practice [8]. Up to present, people still have not come to an identical conclusion as to the research on incompatibility of drugs in prescription [1]. Therefore, study on whether and how Fuzi and Banxia lead to lethal incompatibility could help provide a basis for its clinical application.

It has been well established that *β*-adrenergic receptors (*β*ARs) in the heart play a principal role in regulating cardiac contractility through heterotrimeric G proteins [9]. Recent studies have shown that physiological responses and signal transduction mechanisms of *β*2AR subtype stimulation are distinctly different from those of *β*1AR stimulation [10]. Specifically, *β*2AR activates both Gs (phosphorylation site of serine 346 site, coupled to Gs) and pertussis toxin- (PTX-) sensitive Gi (phosphorylation sites of serine 355, 356, coupled to Gi) protein signaling pathways, whereas *β*1AR is coupled exclusively to the canonical Gs-adenylyl cyclase-cAMP-protein kinase A (PKA) signaling cascade [11–13]. Physiologically, the inotropic response to catecholamine stimulation is mediated mainly by *β*1AR, while the *β*2AR-Gs-mediated cAMP response is suppressed by the coactivated *β*2AR-Gi signaling [14]. Pathologically, in contrast to the predominant role of *β*1AR in heart failure (HF), the balance between toxic versus protective effects for *β*2AR in HF has received great attention [15]. Our previous study revealed that alcohol amine alkaloids of Fuzi exhibited *β*2AR stimulating effect by increasing cAMP levels in HEK 293T cells [16]. In addition, further study in our laboratory had defined PKA downregulation as a crucial event in early cardiac dysfunction by adriamycin (ADR) exposure [17]. However, whether PKA/*β*2AR-Gs/Gi signaling is responsible for interactions of Fuzi-Banxia in ADR-induced cardiomyopathy has yet to be tested.

The goal of this study was to determine whether combination of Fuzi and Banxia is incompatible in ADR-induced cardiomyopathy and to further explore underlying mechanisms by studying the echocardiogram and electrocardiogram (ECG) parameters, chemical markers, myocardial apoptosis, and the *β*2AR-coupled Gs and Gi signaling pathway.

## 2. Materials and Methods

### 2.1. Drug Preparation

*Aconitum carmichaelii* Debx. (Fuzi) and* Pinellia ternate* (Thunb.) Breit. (Banxia) were purchased from Beijing Huamiao Pharmaceutical Co., Ltd. (Beijing, China). Fuzi (Hei Shunpian) and Banxia (Qing Banxia) as well as the combination (1:1) of the two herbs were crushed, respectively, to pieces and then extracted twice with 10 and 8 volumes of boiling water, respectively, for 1h after cold soaking for 0.5h. The combined extract was filtered and subjected to condensation under reduced pressure of 60°C. Finally, per milliliter of the drug concentration was determined to contain 1.35, 1.15, and 0.58g of crude herbs, respectively. The solution was stored at −20°C before being diluted with distilled water for further usage, according to the standard of 0.54g/ml (w/v) or 1.08g/ml.

### 2.2. Experiment Animals

Male Sprague Dawley (SD) rats weighing 250±10g were obtained from the Lab Animal Center, Institute of Health and Environmental Medicine, Academy of Chinese Military Medical Sciences, Tianjin, People's Republic of China [SCXK-(military) 2014-0001]. The rats were housed in a room with a 12 h light and dark cycle and controlled ambient temperature (22±2°C) and humidity (50-60%). They were allowed for ad libitum access to food and water. All the animal experiments were performed in accordance with approved guidelines specified by the animal ethics committee of Tianjin University of Traditional Chinese Medicine (Tianjin, China; no. TCM-LAEC2016035).

### 2.3. ADR-Induced Cardiomyopathy and Animal Treatment

ADR-induced HF was generated as our previous work [17]. ADR was administered intraperitoneally in five equal injections (each containing 3 mg·kg^−1^) over a period of two weeks, with total cumulative dose of 15mg·kg^−1^ body weight. According to the account of Chinese pharmacopoeia document, the adult dose of Fuzi was 3-15g crude drug and the given dosage in rats was 5.4g/kg which was equivalent to 4-fold maximum clinical dose. In treatment group, rats were given low dose Fuzi (FL, 5.4g·kg^−1^·d^−1^), Banxia (BX, 5.4g·kg^−1^·d^−1^), Fuzi and Banxia combination (FB, 5.4g·kg^−1^·d^−1^ + 5.4g·kg^−1^·d^−1^), and high dose Fuzi (FH, 10.8g·kg^−1^·d^−1^) once a day concurrently with ADR during the 2-week period via intragastric administration (n = 15/group). Control and ADR animals received the vehicle (saline) only.

### 2.4. Echocardiography

Transthoracic echocardiographic determinations were conducted in the lateral decubitus position through the visualSonics Vevo 2100 with a 21 MHz transducer (Canada). Echocardiography was performed in anesthetized animals using isoflurane (3%-4% for anesthesia induction; 1%-2% for anesthesia maintenance). Time-motion mode measurements were performed in the parasternal long-axis view. Some of the measured parameters included diastolic/systolic septal wall thickness (IVSd/IVSs), diastolic/systolic posterior wall thickness of the left ventricle (LVPWd/LVPWs), end-diastolic and end-systolic diameter of the LV (LVEDd/LVEDs), and end-diastolic and end-systolic volume of the LV (LVEDV/LVESV). The following parameters were determined: ejection fraction (EF) = (LVEDd – LVEDs) / LVEDd × 100%; LV mass = 1.04 × [(LVEDd + LVPWd + IVSd)^3^ – LVEDd^3^] – 13.6; stroke volume (SV) = LVEDV – LVESV; cardiac output (CO; mL/min) was determined by multiplying HR by SV. For each M-mode tracing, at least three consecutive cardiac cycles were sampled.

### 2.5. Electrocardiogram

The rats were anaesthetized (1% pentobarbital sodium, 50mg/kg), fixed in the supine position, and subjected to ECG for spontaneous breathing. Recordings were made with electrodes in the form of hypodermic needles using a computerized eight-channel ECG analysis module (AD Instruments Pty Ltd., Sydney, Australia) and all the data were analyzed using LabChart 7.3.7 software. The QT interval in milliseconds (ms) was calculated as the time from the beginning of the QRS to the end of the T wave and the rate-corrected QT (QTc) interval was calculated using the Bazett formula: QTc = QT/(RR)^1/2^. The heart rate in beat per minute (bpm) was calculated by dividing 60 by the mean RR interval.

### 2.6. Enzyme-Linked Immunosorbent Assay (ELISA)

Plasma samples (3.8% sodium citrate anticoagulation) applied for an ELISA of brain natriuretic peptide (BNP) and cardiac troponin I (cTn-I) (Shanghai Lianshuo Biological Technology Co., Ltd., Shanghai, China), according to the manufacture's instruments.

### 2.7. Morphological Experiments

For the assessment of histopathological changes, heart tissues fixed with 4% buffered paraformaldehyde were embedded in paraffin, and 3-5*μ*m thick sections were prepared. Sections were then stained with hematoxylin and eosin or Masson trichrome staining as previously described [15].

### 2.8. Apoptosis Detection

Apoptotic nuclei were detected by terminal deoxynucleotidyl transferase dUTP nick end labeling (TUNEL) as previously described [15]. Briefly, paraffin sections of heart tissue were routinely dewaxed, rehydrated, and treated with 3% H_2_O_2_ buffer blocked with endogenous peroxidase followed by 30 min proteolytic digestion in proteinase K (20 *μ*g/ml in 10 mM Tris/HCl, pH 7.4) at 37°C. TUNEL reaction mixture was added dropwise and incubated at 37°C for 60 min in a humidified atmosphere in the dark. A converter peroxidase was added to incubate the slides for 30 min followed by color development at room temperature with 3,3-diaminobenzidine (DAB) substrate. The sections were counterstained with hematoxylin. The TUNEL index (%) was analyzed in 10 randomly random LV heart fields per tissue section using light microscopy (magnification, ×400, Leica Microsystems, Wetzlar, Germany) and calculated as the ratio of the number of TUNEL-positive cells divided by the total number of cells.

### 2.9. Western Blot Analysis

Equal amounts of protein (40 *μ*g) from the LV hearts of rats were separated and electrotransferred onto PVDF membranes (IPVH00010, Merck KGaA), which were probed with rabbit anti-*β*2-AR polyclonal antibody (1:1000, Abcam), rabbit anti-GRK2 polyclonal antibody (1:500, Abcam), rabbit anti-phospho (p)-*β*2-AR polyclonal antibody (pSer346, 1:1000, Sigma-Aldrich), rabbit anti-p-*β*2-AR polyclonal antibody [pSer(355,356), 1:500, Sigma-Aldrich], rabbit anti-PKA polyclonal antibody (1:1000, EMD Millipore), and rabbit anti-tubulin primary antibody (1:500, Santa Cruz). Blots were washed and incubated in peroxidase-conjugated goat anti-rabbit IgG (1:3000, Zhongshan Goldenbridge Bio, Beijing, China). Blots were developed with the enhanced chemiluminescence method (ECL) following the manufacture's instruments (WBKLS0500; Merck KGaA) and then visualized by Vilber Fusion FX7 RT-ECL scanner (Vilber Lourmat, France). The levels of pSer346 and pSer(355,356) from densitometry were normalized to *β*2-AR levels whereas others proteins were normalized to the tubulin levels.

### 2.10. Statistical Analysis

Data are given as mean ± standard deviation. The one-way ANOVA were applied to indicate significantly different mean values for multiple comparisons. A P-value < 0.05 was considered statistically significant.

## 3. Results

### 3.1. Effect of FB on Heart Function

**Figure 1:**
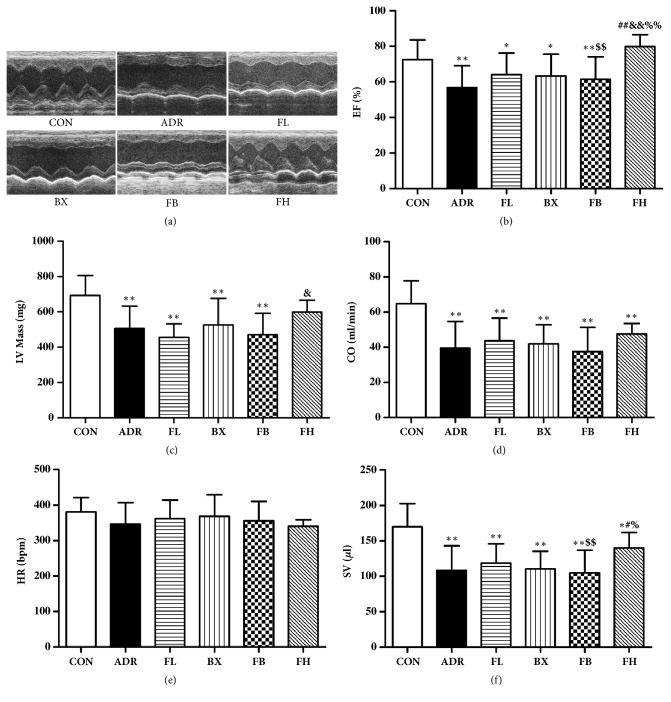
Echocardiogram parameters in ADR rats treated with Fuzi and Banxia combination. The echocardiograms (a) were obtained using the Vevo 2100 high-resolution ultrasound system. Percent ejection fraction (b), left ventricle mass (c), cardiac output (d), heart rate (e), and stroke volume (f) data are also shown in bar groups. ^*∗*^*P*<0.05, ^*∗∗*^*P*<0.01, compared to the control group; ^#^*P*<0.05, ^##^*P*<0.01, compared to the ADR group; ^&&^*P*<0.01, compared to the FL group; ^%^*P* < 0.05; ^%%^*P*<0.01, compared to the BX group; ^$$^*P*<0.01, compared to the FH group. The data are presented as the mean ± SD (n=15 in each group).

As shown in Figure 1, LVEF, LV mass, CO, and SV were notably lower in the ADR model group than in the control group. Compared to other groups, the percent EF in the rats of high dose Fuzi was obviously higher. Similarly, FH treatment significantly enhanced SV compared with ADR model group. However, as to LVEF, LV mass, CO, and SV, no significant difference was observed in ADR-induced rats following treatment with either Fuzi, Banxia, or combination as compared to ADR model group.

### 3.2. Effects of FB on ECG Parameters and Chemical Markers in ADR-Treated Rats

**Figure 2:**
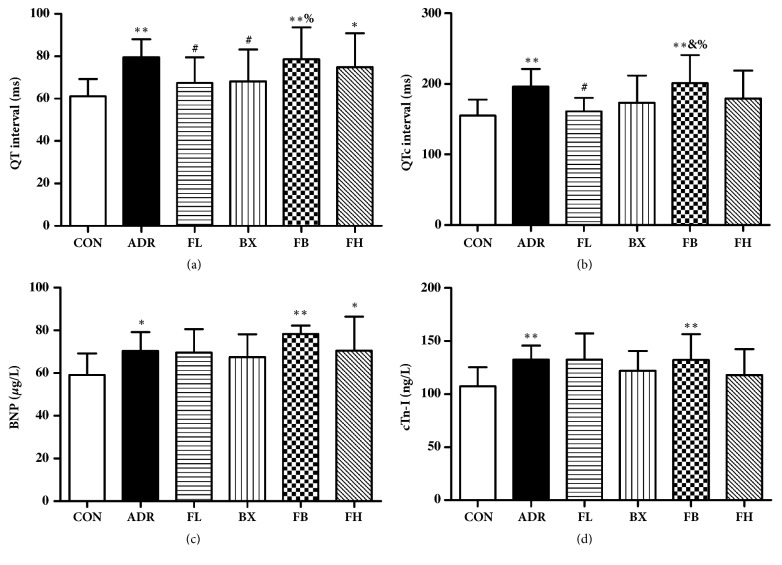
Summary data for electrocardiogram parameters (a and b) and chemical markers (c and d). ^*∗*^*P*<0.05, ^*∗∗*^*P*<0.01, compared to the control group; ^#^*P*<0.05, compared to the ADR group; ^&^*P*<0.05, compared to the FL group; ^%^*P*<0.05, compared to the BX group. The data are presented as the mean ± SD (n=15 in each group).

Analysis of ECG recordings revealed a significant prolongation of QT and QTc intervals, compared with age-matched control rats. As shown in Figures 2(a) and 2(b), treatment with low dose of Fuzi or Banxia alone in ADR-treated rats significantly prevented the ADR-induced QT and/or QTc prolongation. However, the combined treatment of two drugs significantly lengthened QT and/or QTc interval duration compared with Fuzi and/or Banxia alone treatment. Moreover, the activities of plasma BNP and cTn-I elevated markedly in ADR-treated rats as compared to normal control rats (Figures 2(c) and 2(d)). Importantly, FL, BX, FB, and FH pretreatment in ADR-treated rats had no influence on the BNP and cTn-I activities.

### 3.3. Effects of FB on Myocardial Morphological Change and Cardiac Fibrosis

**Figure 3:**
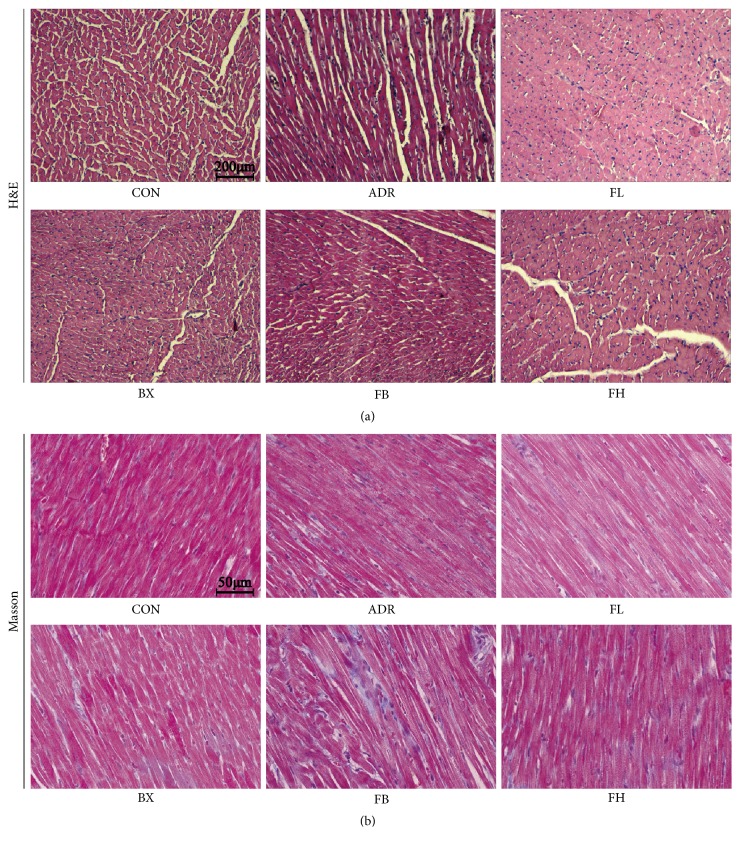
Representative photomicrographs of H&E staining on myocardial morphology (a) and Masson staining on interstitial fibrosis (b). H&E (scale bar, 200*μ*m) and masson (scale bar, 50*μ*m; collagen fibers were stained blue) staining revealed scattered inflammatory cell infiltration and interstitial fibrosis in left ventricle of ADR-induced cardiomyopathy in rats which were not significantly affected by either treatment.

Histopathological study revealed that rats in the control group had normal tissue morphology, while myocardial tissues from the ADR-treated rats exhibited scattered inflammatory cell infiltration (Figure 3(a)). In parallel, under microscope, little amount of collagen was found in normal control rats, whereas there were increasing interstitial and perivascular space in the ventricle of ADR-injured rats (Figure 3(b)). Neither cardiac morphology nor fibrosis was significantly altered by different treatment group.

### 3.4. FB Treatment Increased Apoptosis in ADR-Induced Cardiomyopathy

**Figure 4:**
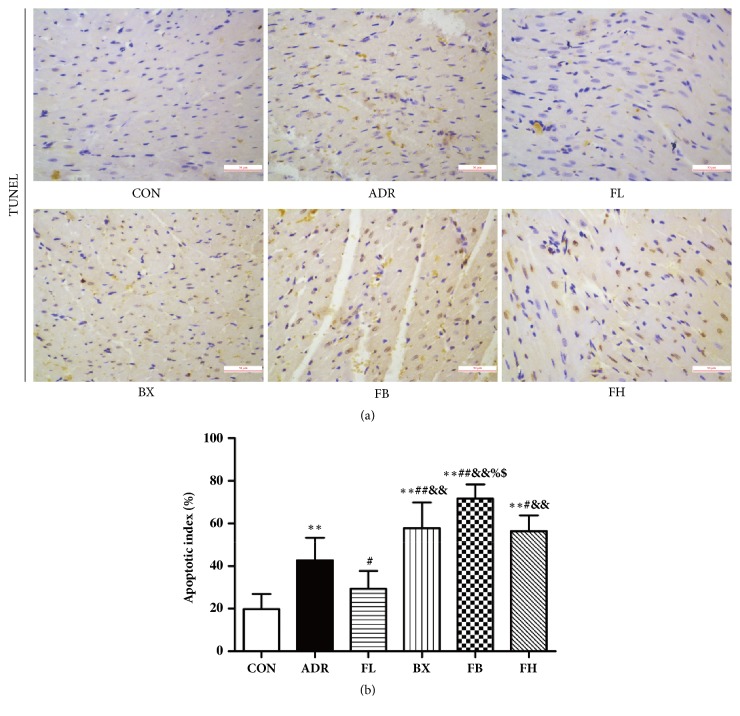
FB increased myocardial cell apoptosis in ADR-induced cardiomyopathy. ^*∗∗*^*P*<0.01, compared to the control group; ^#^*P*<0.05, ^##^*P*<0.01, compared to the ADR group; ^&&^*P*<0.01, compared to the FL group; ^%^*P*<0.05, compared to the BX group; ^$^*P*<0.05, compared to the FH group. TUNEL staining, scale bar, 50*μ*m. The data are presented as the mean ± SD.

The results and subsequent analysis of the TUNEL staining revealed that numbers of apoptotic myocardial cells in the ADR group were significantly higher than those in the normal control group, whereas low dose Fuzi treatment dramatically reduced apoptosis rates (Figure 4). However, the number of TUNEL-positive cells was further significantly enhanced in treatment with BX, FB, or FH for 2 weeks simultaneously with ADR injection as compared to control, ADR, or FL group, respectively. In addition, an increased apoptotic index was observed in ADR rats treated with FB compared with those of BX and FH treatment groups.

### 3.5. Combination of Fuzi and Banxia Treatment against the Effects of Fuzi Alone on *β*2-AR Signaling

**Figure 5:**
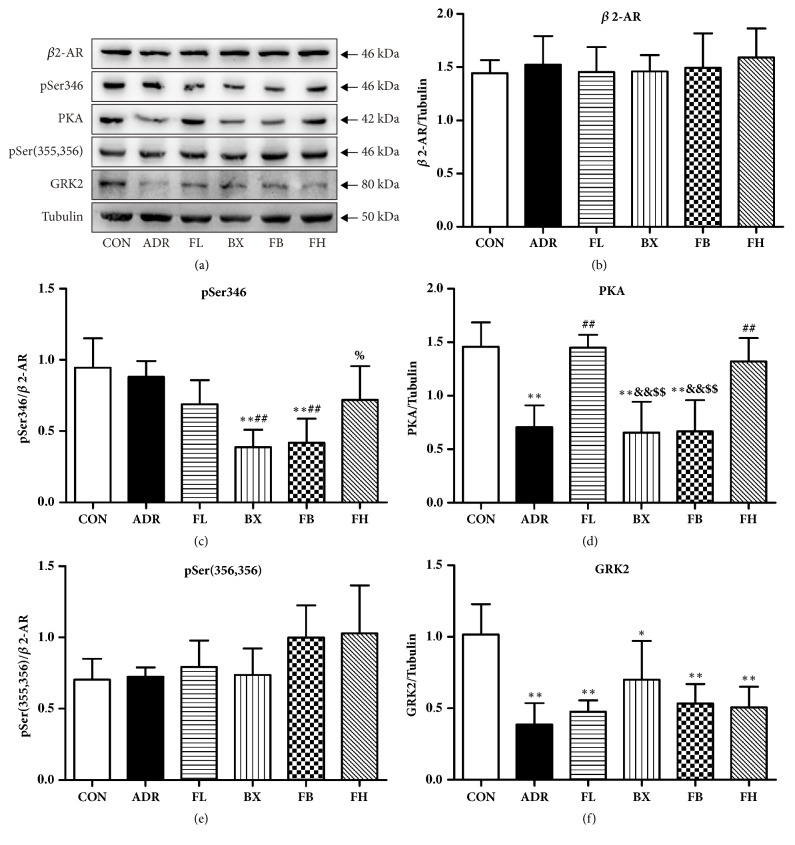
Effect of FB on PKA/*β*2AR-Gs/Gi signaling. The changes of *β*2-adrenergic receptor signaling including *β*2-AR, *β*2-AR phosphorylation statuses pSer346 and pSer(355,356), protein kinase A (PKA), and G protein-coupled receptor kinase2 (GRK2) were all revealed by western blot. ^*∗*^*P*<0.05, ^*∗∗*^*P*<0.01, compared to the control group; ^##^*P*<0.01, compared to the ADR group; ^&&^*P*<0.01, compared to the FL group; ^%^*P*<0.05, compared to the BX group; ^$$^*P*<0.01, compared to the FH group.

As shown in Figures 5(a), 5(b), and 5(e), there were no significant differences in the expression of *β*2-AR and pSer(355,356) among groups. In ADR-injected rats, PKA and GRK2 were significantly decreased in rat as compared to vehicle-injected control rats (Figures 5(d) and 5(f)). Administration of Fuzi with low and high dose effectively restored PKA protein expression (Figure 5(d)). By contrast, no significant difference was observed in the expression of PKA following treatment with combination or BX alone. Moreover, FB and BX treatment significantly reduced the expression level of pSer346 compared with control or ADR group (Figure 5(c)). In addition, all the treatment groups were not normalized for the GRK2 level compared with ADR group (Figure 5(f)).

## 4. Discussion

In the present study, we reported for the first time that the incompatible pairs of Fuzi and Banxia treatment aggravated ADR-induced cardiomyopathy in a rat model. The main findings of this study included that combination of Fuzi and Banxia was detrimental against the improving effect of Fuzi or Banxia alone on ADR-induced prolongation of QT and QTc intervals. Fuzi and Banxia combined treatment greatly increased myocardial apoptosis and the underlying mechanism may be associated with the suppression of PKA/*β*2AR-Gs signaling.

The utility of ADR antineoplastic agent in the clinic is compromised by the risk of cardiotoxicity [18]. Currently, ADR is largely used to induce cardiomyopathy and HF. The drug was injected intraperitoneally as a cumulative dose of 15 mg/kg in a 2-week period was suggested for ADR-induced HF onset in rats [19]. Evidence from our previous experiment has demonstrated a significant reduction of cardiac systolic function in ADR-induced cardiomyopathy at the injection endpoint (2 weeks), which was accompanied by low levels of cardiomyocyte apoptosis and marked inhibition of PKA expression [17]. Similarly, our present study obtained consistent findings.

Nowadays, Fuzi is widely used in the treatment for HF due to lots of efficacy such as improving the myocardial contraction, dilation of blood vessels, and increasing the blood flow [20]. Consistently, our experiments reported a significant relief of ADR-induced heart dysfunction following high dose Fuzi treatment, as indicated by elevated percent EF and SV as well as slight increased trend of LV mass. By contrast, either Fuzi, Banxia, or their combination had no effect on cardiac function, indicating that the positive inotropic effect is not affected when the herb pair is combined together. It has been suggested that acute QT prolongation plays a key role in ADR-induced cardiotoxicity [18]. Our findings were in agreement with the previously published reports and revealed that there was a marked reduction in QT and/or QTc prolongation in low dose of Fuzi and Banxia alone groups compared to the ADR group. However, no significant difference was observed in the ADR-induced QT and QTc interval prolongation following combination of the two drugs treatment, suggesting that Fuzi and Banxia interaction attenuated the protective effect of each drug alone. This can be interpreted as an incompatible situation where the therapeutic effect of one herb is diminished by another herb. Collectively, these results indicated that combination of Fuzi and Banxia antagonized therapeutic effect of each drug alone when used at ADR-induced cardiomyopathy rat model.

Multiple mechanisms are involved in ADR-induced HF. ADR-induced cardiomyopathy is strongly linked to an increase in cardiac oxidative stress, which subsequently activates apoptotic signaling leading to cardiomyocyte apoptosis [21], whereas it was previously reported that four alkaloid components from Fuzi were screened and considered as specific active components that counteract ADR-induced HF [22]. On this basis, the present study suggested that the increased apoptotic index by ADR exposure was significantly decreased by FL, indicating the potential of FL for inhibition of apoptosis. In contrast to the antiapoptotic effect of FL, FB treatment substantially elevated the intensity of apoptosis. These results suggested that Fuzi and Banxia combination treatment could trigger apoptosis against protective effect of Fuzi on ADR-induced rat model. This detrimental effect of FB may be mainly arising from proapoptotic response mediated by Banxia, which significantly increased cardiomyocyte apoptosis compared to ADR group; others have previously demonstrated that excessive or long-term use of this herb can cause cardiotoxicity [23]. Indeed, the given dosage 5.4g/kg (equivalent to 60 g crude drug per person per day) of Banxia in the present study was far superior to acceptable standards laid down by the Chinese pharmacopoeia (3-9 g per person per day). It is worthy of mention that cell apoptosis was decreased in FL while increased in FH condition as compared to ADR model group, respectively. Similarly, from the above ECG analysis, it is also important to note that ADR induced QT interval lengthening was reduced by FL treatment but not significantly changed by FH treatment. The dual-directional regulation effects of TCM from the composition may help to explain the opposite effects resulting from FL or FH treatment [24]. Specially, TCM consists of complex chemical compositions, which paints a picture of great complexity and multiple effects through acting on various receptors/enzymes in the body as the proportion changes of the active ingredients. Hence, a single drug may produce different or opposite effects due to the pharmacologically antagonistic components contained in the same TCM. Indeed, it has been reported that four compounds (talatizamine, 14-acetyl-talatizamine, hetisine, and 14-benzoylneoline) from Fuzi could counteract ADR-induced HF [22], whereas aconitine from Fuzi may induce apoptosis in H9c2 cells [25]. At the same time, Fuzi creates antagonistic chemical compositions, exerting antiarrhythmic effect (N-sepaconitine, N-deacetyllappaconitine, ranaconitine, lappaconitine, 14-acetyltalatisamine, sepaconitine, etc.) or proarrhythmic effect (aconitine, mesaconitine, hypaconitine, etc.), respectively [26]. Therefore, for FL or FH extracts, different composition ratio of active ingredients may result in different or antagonistic effects as presented in our study.

It is now generally accepted that, as the major effector of cAMP, PKA is the master regulator of cardiac muscle contraction [9]. We previously reported that ADR-treated rats revealed a marked reduction of PKA and that this too was repeated in here. Thus, consistent with restored effects of PKA on heart function, here we displayed that high dose Fuzi treatment significantly enhanced LV performance, which occurred concurrently with strong expression of PKA. Indeed, our previous study had confirmed that Fuzi could activate *β*2AR by increasing cAMP levels in HEK 293T cells [16]. However, FB and BX treatment attenuated Gs-biased *β*2AR signaling, which indicated that cardiac inhibition of FB was more likely related to Banxia against the inotropic response of Fuzi, suggesting further the incompatibility of Fuzi and Banxia in ADR-induced cardiomyopathy. On the other hand, GRK2 has been implicated as a causal factor of HF in various experimental models [27], but its role in ADR induced HF is unknown until now. In this study, however, GRK2 level was decreased following exposure to the ADR condition and no significant difference was observed in each treatment group. Moreover, since previous studies have reported that GRK2 is upregulated in the context of oncogenic signaling [28], it is tempting to suggest that inhibition of GRK2 expression might be involved in the anticancer effect of ADR. Additionally, given that the *β*1AR is by far the predominant AR subtype in the heart, where it promotes cardiac contractility, it comes as no surprise that most studies have focused on associations of the *β*1AR with HF [29–32]. Indeed, our previous study has indicated that downregulation of *β*1AR is associated with ADR-induced cardiomyopathy [17]. However, the effects of Fuzi-Banxia herbs on the signaling and function of this AR subtype have failed to be confirmed in the present study. Of course, these associations await further confirmation.

Importantly, a recent study demonstrated that blocking directly the PKA pathway almost completely prevented cancer cell growth, especially of detached anoikis-resistant cells, indicating that PKA is a potential chemotherapeutic target to prevent cancer cell metastasis [33]. In fact, ADR is used as a common chemotherapeutic agent and our experiments supported that the proapoptosis effect of ADR was robustly related to the suppression of PKA whereas restored PKA expression in FL group attenuated cardiac cell apoptosis. Similarly, aggravated apoptosis in BX and FB group was also associated with inhibition of PKA expression. Form this point of view, the present study provided scientific rational of the potential utility value of combination of Fuzi and Banxia for anticancer except for cardiotoxicity. Indeed, either Fuzi or Banxia has been widely used for its various pharmacological effects, such as antitumor, anti-inflammatory, and analgesic properties. Moreover, the incompatible pair has also been prescribed for treating incurable diseases [6, 7]. However, further studies are required to prove this hypothesis.

## 5. Conclusions

Combination of Fuzi and Banxia antagonized the cardioprotection of Fuzi on ADR-treated rats, which might be related to the prolongation of QT and QTc intervals, the promotion of cardiac cell apoptosis, and inhibition of Gs-biased *β*2AR signaling. In conclusion, the present study provided scientific rational for incompatibility of Fuzi and Banxia, which helps to understand the risks and limitations when using incompatible pairs in clinical applications.

## Data Availability

The data used to support the findings of this study are available from the corresponding author upon request.
